# A Fast and Sensitive Luciferase-based Assay for Antibody Engineering and Design of Chimeric Antigen Receptors

**DOI:** 10.1038/s41598-020-59099-9

**Published:** 2020-02-11

**Authors:** Venkatesh Natarajan, Ramakrishnan Gopalakrishnan, Hittu Matta, Sunju Choi, Songjie Gong, Alberto Jeronimo, Pooja Smruthi Keerthipati, Anthony Morales, Harishwar Venkatesh, Preet M. Chaudhary

**Affiliations:** Jane Anne Nohl Division of Hematology and Center for the Study of Blood Diseases, University of Southern California, Keck School of Medicine, Los Angeles, California, United States of America

**Keywords:** Applied immunology, Cancer

## Abstract

Success of immunotherapeutic approaches using genetically engineered antibodies and T cells modified with chimeric antigen receptors (CARs) depends, among other things, on the selection of antigen binding domains with desirable expression and binding characteristics. We developed a luciferase-based assay, termed Malibu-Glo Assay, which streamlines the process of optimization of an antigen binding domain with desirable properties and allows the sensitive detection of tumor antigens. The assay involves a recombinant immunoconjugate, termed Malibu-Glo reagent, comprising an immunoglobulin or a non-immunoglobulin based antigen binding domain genetically linked to a marine luciferase. Malibu-Glo reagent can be conveniently produced in mammalian cells as a secreted protein that retains the functional activity of both the antigen binding domain and the luciferase. Moreover, crude supernatant containing the secreted Malibu-Glo reagent can directly be used for detection of cell surface antigens obviating the laborious steps of protein purification and labeling. We further demonstrate the utility of Malibu-Glo assay for the selection of optimal single chain fragment variables (scFvs) with desired affinity characteristics for incorporation into CARs. In summary, Malibu-Glo assay is a fast, simple, sensitive, specific and economical assay for antigen detection with multiple applications in the fields of antibody engineering, antibody humanization and CAR-T cell therapy.

## Introduction

Antibodies are arguably the most versatile class of biologics with wide applications in basic research, disease diagnosis and therapy. Besides full-length antibodies, advances in antibody engineering have led to the generation of minimal antigen binding fragments (e.g., Fab), single chain fragment variable (scFv), diabodies, bispecific and single domain antibodies (sdAbs)^1,2^. These antibodies are further optimized by mutagenesis, CDR grafting, affinity maturation and humanization to generate a large number of variants. Altogether, these efforts have increased the pool of antibodies against specific therapeutic targets. However, the challenge of identifying an optimal antibody with desired characteristics for a specific application remains. Robust assays to narrow down the number of antibody candidates for downstream evaluation are critically needed to avoid wasteful preclinical research efforts.

Immunotherapy applications of antibodies have extended beyond their use as soluble biological agents. In recent years, scFvs derived from antibodies have been grafted onto T cell receptors to create Chimeric Antigen Receptor (CAR) that can be used to redirect immune cells to desired cancer cells^3,4^. For this purpose, scFvs that fall within an optimum affinity range are desired^5,6^. CAR-T cells with high affinity scFvs are reported to exhibit robust antitumor efficacy, but it is accompanied by profound off-tumor-on-target toxicity against normal cells expressing low levels of antigen^7,8^. Additionally, CAR-T cells containing extremely high affinity scFv often result in tonic signaling^8^. By contrast, CAR-T cells designed using low affinity scFvs are more tumor-selective with minimal off-tumor toxicity^6,9^. Thus, depending on the relative antigen density of tumor *vs* normal cells, affinity of scFv has to be fine-tuned to generate an optimal CAR^10–12^. Equally important, scFvs that are used in the construction of CARs should express well. Thus, development of a reliable and simple method to screen an existing pool of scFvs based on their expression and affinity would expedite CAR-T cell engineering.

Although, several methods (*e.g*., ELISA, immunoblotting, flow cytometry and surface plasma resonance *etc*.) are currently available to detect binding to a target antigen^13^, only a few methods (e.g., flow cytometry and AlphaLISA) are suitable for detecting antibody binding to antigen in its native conformation on the cell surface^14^. Invariably, all of these assays rely on a detection tag, linked either directly to a primary antibody, a secondary antibody or other proteins that recognize the primary antibody^15^. These detection tags historically range from radioisotopes, small molecule fluorophores, fluorescent proteins or enzymes. In general, chemical methods are used to conjugate an antibody to a desired protein or a fluorophore. However, these chemical crosslinking reactions are difficult to control resulting in heterogeneous mixture with variable stoichiometry. Further, such conjugation may alter the biological activity of antibody. These limitations can be circumvented using recombinant DNA technology that allows genetic fusion of an antibody to a desired protein. For instance, antibodies and antibody fragments fused to reporter enzymes such as alkaline phosphatase^16^ or beta-galactosidase^17^ have been developed. However, their widespread use has been hampered by the larger size of these enzymes and limited sensitivity.

Luciferases are versatile tools in biomedical research as they combine high sensitivity with low background^18,19^. Although Firefly luciferase (Fluc) is one of the most popular luciferase for research applications, its large size (61 kDa) has hampered its use in protein fusion studies. In contrast to Fluc, marine luciferases and their engineered derivatives are smaller in size (approximately 19 kDa) and possess greater brightness, making them ideal partners for fusion studies^18,20^.

In this report, we describe a marine luciferase based assay for antigen detection. As the assay utilizes marine luciferase, we named it Malibu-Glo Assay after the Malibu Beach in California. We show that marine luciferases can be fused to scFvs, single domain antibodies (vHH) and antibody-like scaffolds (Centyrins) to yield versatile Malibu-Glo detection reagents. We demonstrate the utility of the Malibu-Glo assay to rapidly evaluate the relative expression and binding capabilities of different scFv variants. We further demonstrate the utility of the assay for the selection of optimal scFv for incorporation into CARs.

## Results

### Design and construction of Malibu-Glo reagent (scFv-Nluc fusion) expression constructs

**Figure 1:**
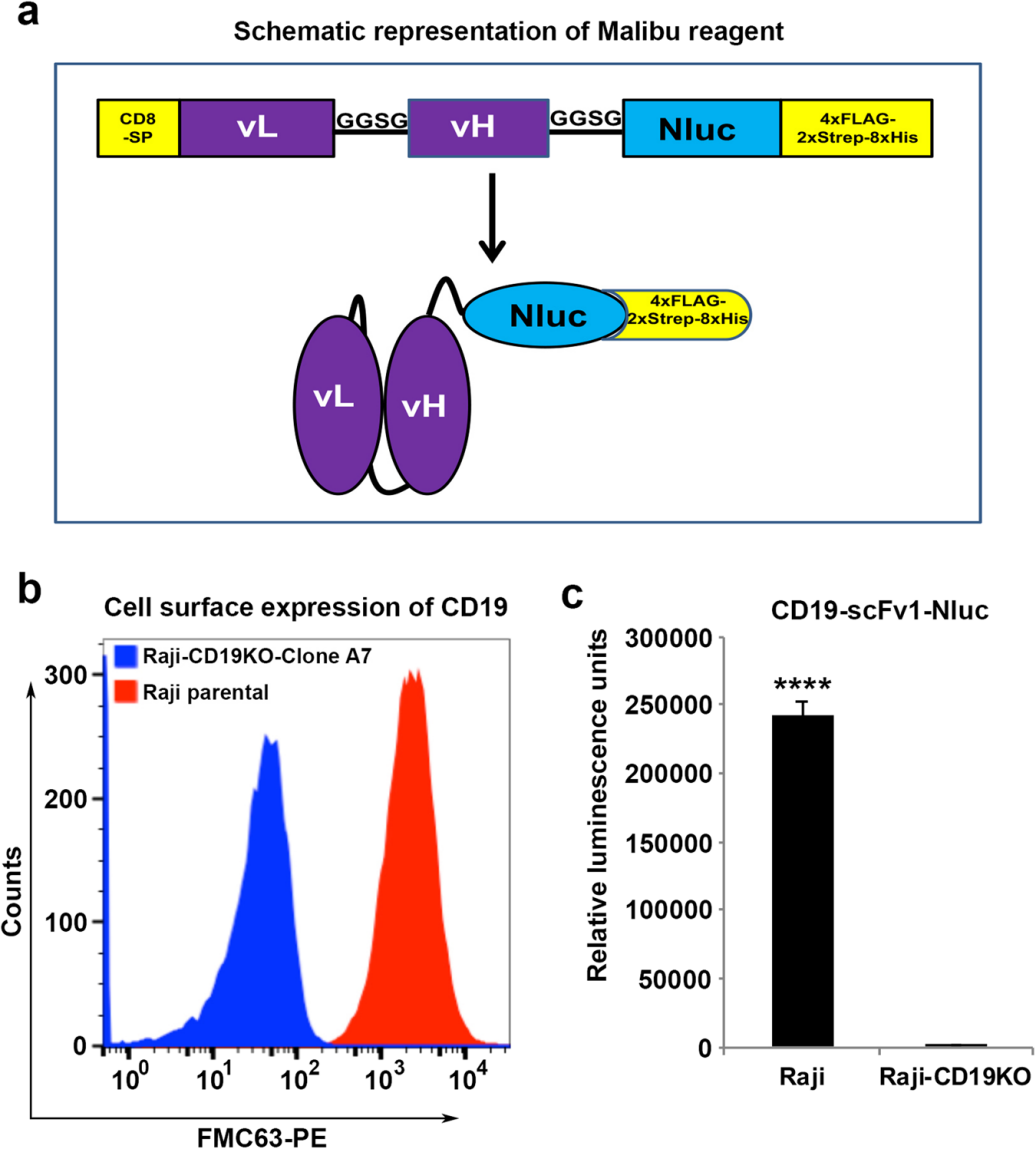
Generation and Characterization of Raji cell clones lacking CD19. **(a)** Schematic representation of Malibu-Glo (scFv-Nluc) construct **(b)** Lack of CD19 expression in Raji-CD19KO clone A7 was confirmed by staining with PE conjugated FMC63 antibody followed by flow cytometry. **(c)** Lack of CD19 expression in Raji-CD19KO clone A7 was confirmed by Malibu-Glo assay. Raji cells or Raji-CD19KO clone A7 (2 × 10^5^ in 100 µl) were incubated with 100 µl of supernatants containing CD19-scFv1-Nluc fusion protein (Malibu-Glo reagent) for 45 minutes on ice. Then, cells were washed 5 times with PBS containing 0.5% FBS prior to measurement of luminescence associated with cell pellet as described in *Materials and Methods*. Statistically significant differences were shown by asterisks (****) at a level of *P* < 0.0001.

To develop a luciferase based assay for antigen detection (Malibu-Glo assay), we generated a lentiviral vector encoding a scFv derived from murine CD19 antibody FMC63 in fusion with Nluc via an intervening short Gly-Gly-Ser-Gly flexible linker (Fig. 1a). The resulting fusion protein (Malibu-Glo reagent) was designated CD19-scFv1-Nluc. We selected Nluc for the assay because of its brightness, ATP-independence, small size, monomeric nature, glow type luminescence, and improved stability^21^. In order to facilitate the purification of the Malibu-Glo reagent and allow its detection via flow cytometry, we also added a cassette 4xFLAG-2xStreptag-8xHis to the carboxyl-terminus of the fusion protein via an intervening Glycine-Serine (GGSG) linker. A schematic of the Malibu-Glo reagent (i.e. scFv-Nluc) construct is depicted in Fig. 1a.

### Development of Raji cells lacking CD19 using CRISPR/Cas9 technology

To demonstrate the specific binding of the scFv-Nluc fusion protein to CD19, we generated Raji cells lacking CD19 using CRISPR/Cas9 technology. We successfully isolated 6 clones that completely lacked CD19 expression as determined by staining with CD19 (FMC63-PE) antibody. From these clones, we selected clone A7 (Fig. 1b), termed Raji-CD19KO clone-A7, to serve as a negative control for all binding experiments involving CD19-scFv-Nluc fusion protein.

### Expression and functional evaluation of CD19-scFv1-Nluc fusion protein

The expression construct encoding CD19-scFv1-Nluc fusion protein was transiently transfected into 293 FT cells and supernatant containing the secreted Malibu-Glo reagent was collected 48 hours post transfection and used to bind parental Raji cells and Raji-CD19KO clone-A7. As shown in Fig. 1c, the Raji cells showed over 200-fold increase in luminescence when bound by the CD19-scFv1-Nluc Malibu-Glo reagent as compared to the Raji-CD19KO clone-A7 cells. These results demonstrate that crude supernatant containing the Malibu-Glo reagent generated in mammalian expression system can successfully be employed for specific detection of a cell surface antigen using a widely available luminometer.

### Functional evaluation of Malibu-Glo reagents generated using different marine luciferases

**Figure 2:**
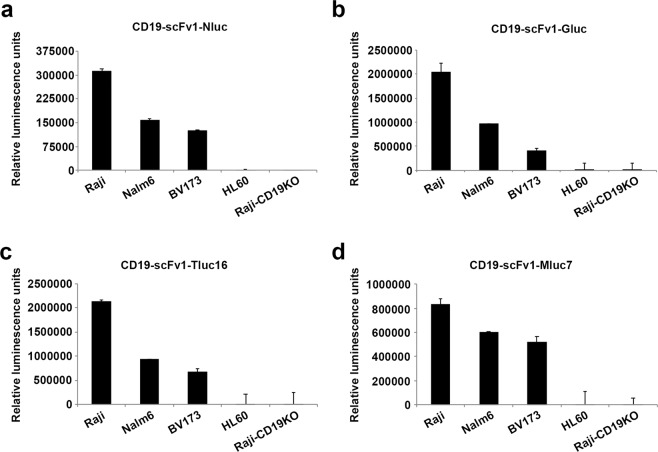
Functional evaluation of Malibu-Glo reagents generated using different marine luciferases. Indicated CD19 expressing cells or control cells lacking CD19 (2 × 10^5^ in 100 µl) were incubated with 100 µl of indicated CD19-scFv1 Malibu-Glo reagents generated using different luciferases for 45 minutes on ice. Then, cells were washed 5 times and the luminescence associated with cell pellet was measured as described in *Materials and Methods*.

In addition to Nluc, a number of marine luciferases, such as Gluc, Tluc16, and Mluc7 have been described^20,22^. To test whether the Nluc based Malibu-Glo assay is extendable to other marine luciferases, we swapped Nluc in the original CD19-scFv1-Nluc fusion construct with Gluc, Tluc16, and Mluc7. These Malibu-Glo reagents were tested for their ability to detect cell surface expression of CD19. In addition to Raji, we evaluated the binding of CD19-scFv1-luc Malibu-Glo reagents to Nalm6 and BV173 (CD19^+ve^ leukemia cell lines). We also included HL60 (CD19^−ve^ acute myeloid leukemia cell line) and Raji-CD19KO-A7 cells as negative controls. All CD19-scFv1-luc Malibu-Glo reagents showed specific binding only to the CD19-expressing Raji, Nalm6 and BV173 cell lines with insignificant or no binding to HL60 and Raji CD19KO-A7 control cell lines that lacked CD19 expression (Fig. 2a-d). Furthermore, all four CD19-scFv1-luc Malibu-Glo reagents showed the same order (Raji > Nalm6 > BV173) of binding in the CD19^+ve^ cell lines. These results demonstrate that irrespective of the type of marine luciferase fused to scFv, our expression system allows for optimal folding that retains both the enzymatic activity of luciferase and the binding specificity of scFv. Thus, in principle, Malibu-Glo assay can be developed using scFv fused to any marine luciferase.

### Malibu-Glo assay allows for rapid screening of scFvs based on their expression and target binding

**Figure 3:**
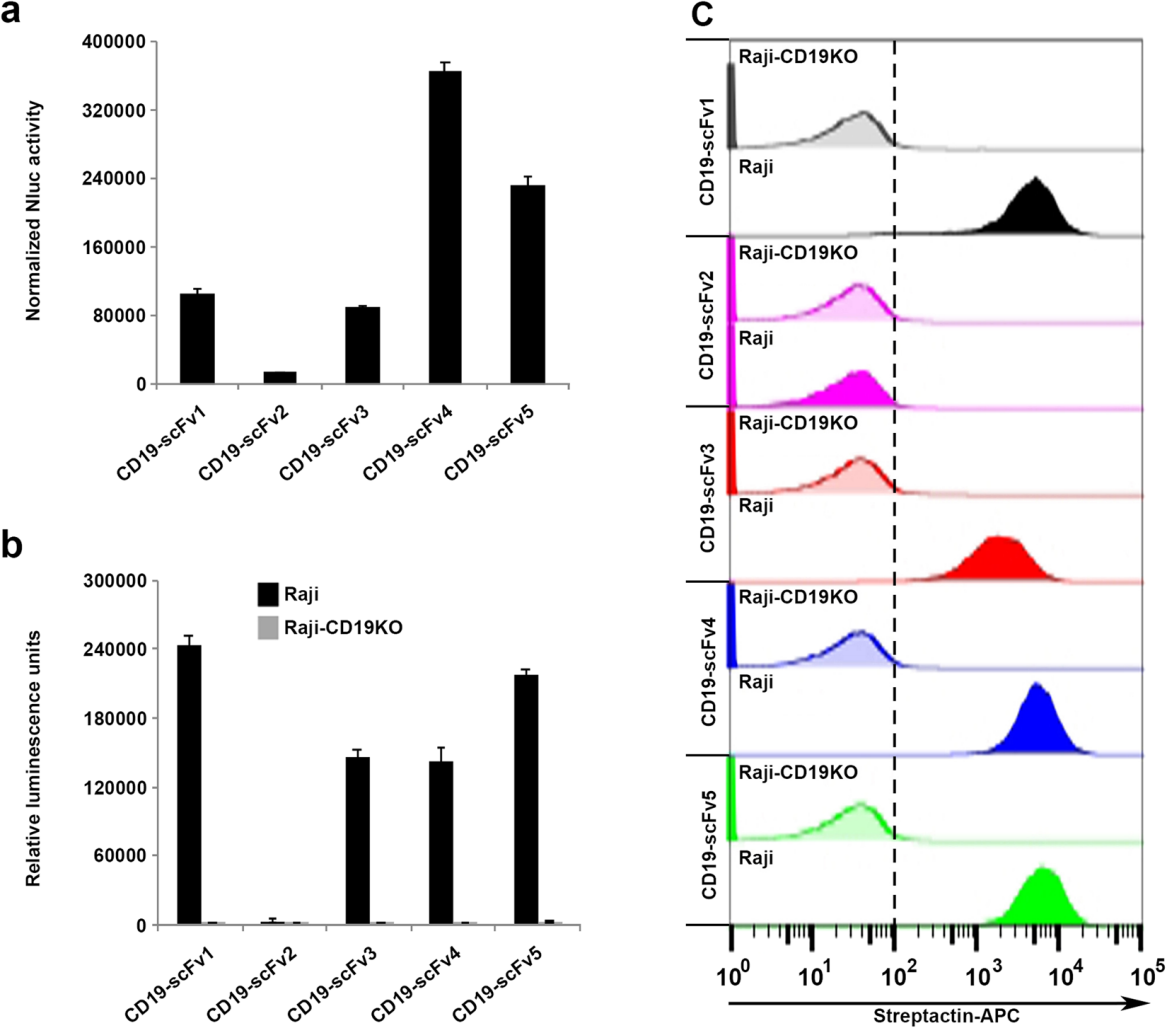
Malibu-Glo assay enables evaluation of expression and target binding characteristics of different CD19-scFvs. **(a)** 293FT cells were transiently transfected with different CD19-scFv-Nluc Malibu-Glo constructs along with the control Fluc construct. After 48 hours, Nluc activity was measured in diluted supernatants (1:100 in PBS containing 0.5% FBS). Firefly luciferase activity was measured in the cell lysates using D-luciferrin as substrate. Nluc luminescence value of each supernatant was normalized with corresponding Fluc luminescence value to control for differences in transfection efficiency. **(b)** Target binding of different CD19-scFvs were assessed by Malibu-Glo assay. Raji and Raji-CD19KO cells were incubated with supernatants containing equivalent luminescence units of indicated CD19-scFvs in a final volume of 200 µl for 45 minutes on ice. Cells were washed 5 times and bound luminescence was measured as described in *Materials and Methods*. **(c)** Target binding of different CD19-scFvs analyzed by flow cytometry. Raji cells and Raji-CD19KO cells were incubated with 100 µl supernatant containing the indicated CD19-scFv-Nluc Malibu-Glo reagents for 45 minutes on ice. After incubation, cells were washed 2 times and incubated with streptactin-APC for 45 minutes. After 2 washes, cells were analyzed by flow cytometry. A representative of two independent experiments was shown.

A major challenge in antibody engineering projects is to select an optimal therapeutic antibody that not only binds to the desired target and exhibit the desired functional activity, but is also well expressed in mammalian cells. As fusion to Nluc allows measurement of luminescence in crude supernatants without the need for laborious and time-consuming steps of protein purification and labeling, we examined whether Malibu-Glo assay can serve as a versatile tool to rapidly screen scFvs based on their relative level of expression. To this end, we generated Nluc fusion constructs of a panel of scFvs directed against CD19. We co-transfected 293FT cells with each scFv-Nluc fusion construct along with an intracellularly expressed firefly luciferase construct to normalize for protein expression. Supernatants containing Malibu-Glo reagents were collected at 48 hours post-transfection and used for the measurement of Nluc luminescence. The firefly luciferase activity was measured in cell lysates and used to normalize Nluc values of the Malibu-Glo reagents present in the supernatants to control for the differences in transfection efficiency. The normalized Nluc activity was used to grade the scFvs based on their expression. As shown in Fig. 3a, CD19-scFv4 and CD19-scFv5 showed high expression, CD19-scFv1 and CD19-scFv3 showed intermediate expression, while CD19-scFv2 showed the lowest expression. The results of the Malibu-Glo assay were confirmed by analyzing the expression of the different CD19-scFv fusion proteins by western blotting using a Flag antibody (Supplementary Fig. 1). The western blot further demonstrated the absence of any truncated proteins and confirmed that only full-length Malibu reagents are being produced. Thus, Malibu-Glo assay offers a simple solution for elimination of poorly expressed scFvs at an early stage of antibody discovery, thereby avoiding futile investment of time and labor in downstream processes.

We next examined if the crude supernatant containing the secreted Malibu-Glo reagents can be used to rapidly assess the relative binding-affinities of different scFvs to their target antigen-expressing cells, thereby obviating the laborious step of protein purification. However, the difference in the expression level of different scFvs posed a problem in the use of the crude supernatant in such an assay. To ensure that the volume of supernatants used in the binding assays contains equal amounts of different scFv-Nluc fusion proteins, we took advantage of the 1:1 stoichiometric relationship between the antigen binding module (i.e., scFv) and the detection module (i.e., Nluc). Therefore, we used luminescence values as a surrogate for the amount of the scFv-Nluc fusion proteins present in the different supernatants and used these values to normalize the volume of the different supernatants used in the binding assay. To test this approach, we used supernatant volumes containing equivalent luminescence units of each scFv in the assay to check their binding to Raji cells and Raji-CD19KO clone A7. As shown in Fig. 3b, CD19-scFv1, CD19-scFv3, CD19-scFv4 and CD19-scFv5 showed strong binding to Raji cells, while CD19-scFv2 showed negligible binding. None of the scFvs showed significant binding to Raji-CD19KO clone A7, confirming the specificity of the assay. To validate the results obtained with the Malibu-Glo assay, we measured the binding of different scFvs to Raji cells by flow cytometry using Strep-Tactin-APC, which binds with high affinity to StreptagII present on the Malibu-Glo reagents (Fig. 3c). Consistent with the results obtained with the Malibu-Glo assay performed using a luminometer, CD19-scFv1, CD19-scFv3, CD19-scFv4 and CD19-scFv5 showed strong binding to Raji cells when measured using flow cytometry, while CD19-scFv2 showed negligible binding.

### Malibu-Glo assay can be used to evaluate expression and relative binding affinities of different humanized scFv variants

**Figure 4:**
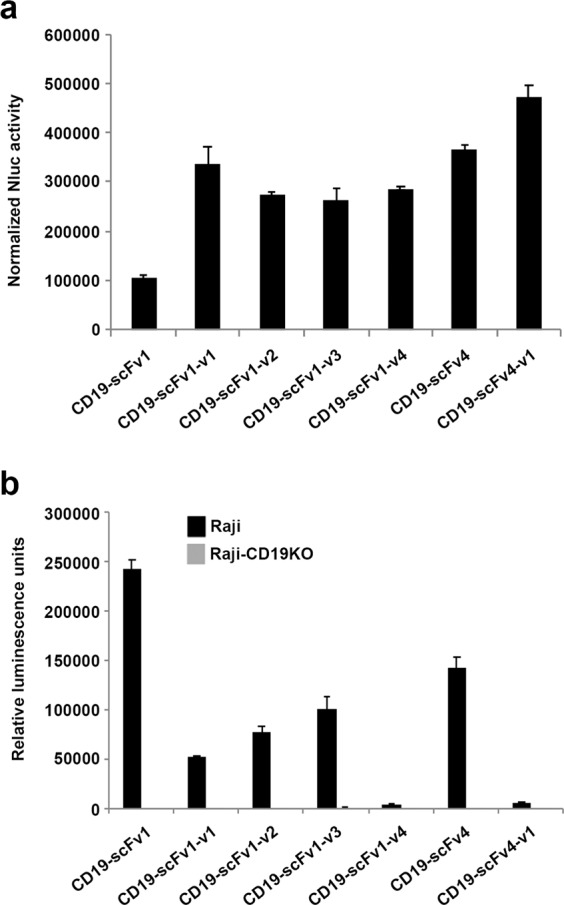
Malibu-Glo assay enables evaluation of expression and target binding of different humanized CD19-scFv variants. **(a)** Expression of different humanized CD19-scFvs and **(b)** binding to Raji cells were evaluated as described for Fig. 3a,b respectively.

Humanization of antibodies and antibody fragments is frequently used in the development of therapeutic antibodies to circumvent the problems caused by immunogenicity. However, a large number of humanized antibody variants have to be screened to ensure optimal protein expression and target-binding. Compared to full-length antibodies, humanization of scFvs poses even a greater challenge due to their lower stability, compromised avidity and poor yields due to aggregation. To test the utility of the Malibu-Glo assay for the selection of humanized scFv variants, we generated Nluc fusion proteins comprising four humanized variants of CD19-scFv1 designated CD19-scFv1-v1 to CD19-scFv1-v4, and one variant of CD19-scFv4 designated CD19-scFv4-v1. The scFv-Nluc fusion constructs were transiently transfected in 293FT cells and relative expression of the secreted fusion proteins in the cell supernatant was examined as described in the preceding section. All four humanized CD19-scFv1 variants expressed well relative to the parental CD19-scFv1, which showed moderate expression (Fig. 4a). Similarly, CD19-scFv4-v1 showed slightly better expression as compared to the parental CD19-scFv4. The results were confirmed by western blotting using a Flag antibody (Supplementary Fig. 2). Next, the supernatants containing the different scFv-Nluc fusion proteins were evaluated for binding to Raji and Raji-CD19KO cells. As shown in Fig. 4b, the CD19-scFv1-v1, CD19-scFv1-v2 and CD19-scFv1-v3 variants showed lower binding to Raji cells as compared to the parental CD19-scFv1, while the CD19-scFv1-v4 variant showed a near complete loss of target-binding. In contrast, although the parental CD19-scFv4 showed slightly lower expression compared to CD19-scFv4-v1, its binding to Raji cells was 22-fold higher. Notably, none of the variants bound Raji-CD19KO cells, thereby demonstrating that they retain their specificity for CD19. Taken collectively, these results demonstrate that Malibu-Glo assay can be used to rapidly determine the expression level, target antigen binding ability and specificity of scFv variants using crude supernatants.

Malibu reagents carry the epitope tags (4xFLAG-2xStreptagII-8xHis) at their C-termini. We purified the CD19-scFv1-Gluc Malibu reagent by affinity chromatography using a Strep-Tactin column^23^. The purified Malibu reagent was used to determine the half-maximal effective concentration (EC50) for binding to 1 × 10^4^ Raji and RS411 cells. Using the variable slope model, we determined that the EC50 of CD19-scFv1-Gluc for binding to Raji and RS411 cells to be 4.9 × 10^−9^ M and 4.7 × 10^−9^ M, respectively (Supplementary Fig. 3).

### Malibu-Glo assay allows for selection of optimal scFvs for CAR design

**Figure 5:**
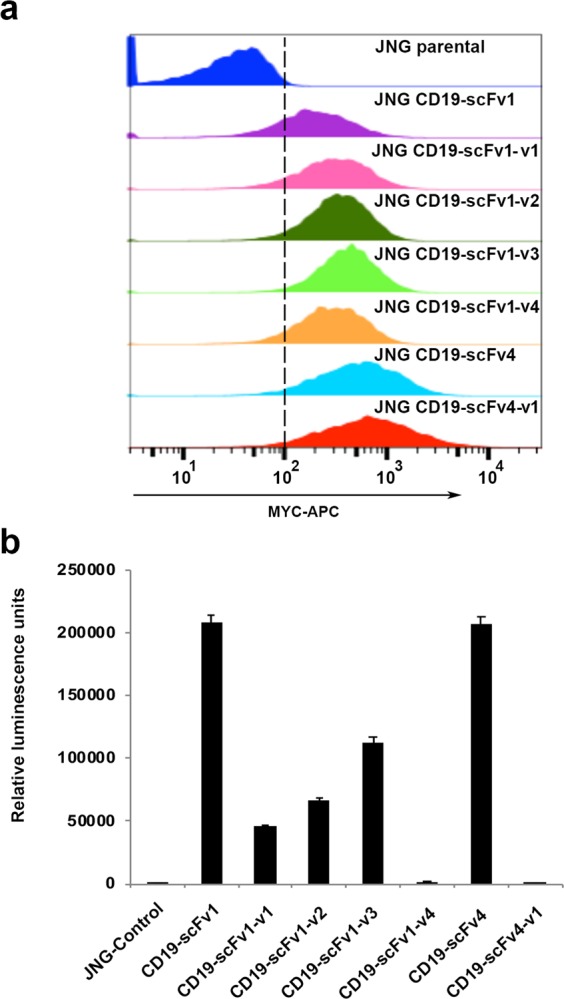
Expression level and binding ability of scFv measured by the Malibu-Glo assay is predictive of the expression and the target binding affinity of the corresponding CAR incorporating that scFv. **(a)** JNG cells stably expressing the indicated MYC-tagged CAR constructs were stained with MYC-APC conjugate and analyzed by flow cytometry to evaluate CAR expression. **(b)** JNG cells stably expressing the indicated CD19-specific CAR constructs were incubated with supernatants containing CD19-ECD-Nluc fusion protein for 45 minutes on ice. After washing steps, cells were assessed for luminescence using Topanga Assay.

To further demonstrate that scFvs selected based on expression levels and target binding using Malibu-Glo assay can aid the choice of scFvs for CAR-T engineering, we generated second generation (BBz) lentiviral CAR constructs based on CD19-scFv1, CD19-scFv4 and their humanized variants. The scFvs were fused in frame to a cassette encoding a MYC epitope tag, a CD8 hinge domain, a CD8 transmembrane domain, a 41BB co-stimulatory domain and a CD3ζ activation domain. A Jurkat cell clone expressing an NFAT-GFP reporter (JNG) was transduced with lentivirus encoding the different CD19-CARs. After 10 days of selection in puromycin, surviving cells were stained with anti-MYC-APC and analyzed by flow cytometry to quantitate CAR expression on cell surface. Consistent with the expression of scFv-Nluc fusion proteins as measured by Malibu-Glo assay (Fig. 4a), all CARs showed relatively good expression in JNG cells (Fig. 5a). To examine if the scFv–Nluc fusion is also predictive of CAR binding to the target antigen, we tested the ability of JNG cells expressing different humanized CARs described above to bind the target antigen CD19. For this purpose, we employed Topanga assay. This assay was recently developed in our lab for the detection of CAR and utilizes CD19-extracellular domain (ECD)-Nluc fusion protein^23^. Equal number of JNG cells expressing the different CD19-CAR were incubated with supernatants containing CD19-ECD-Nluc for 45 min on ice, washed and cell associated luminescence was measured. As shown in Fig. 5b, binding of CD19-ECD-Nluc to JNG expressing different CD19-scFv1-CARs correlated well with the binding of the corresponding scFv-Nluc to Raji cells observed in Fig. 4b. Thus, the CD19-scFv1-CAR showed the highest binding, the CD19-scFv1-v4-CAR showed nearly negligible binding while all the CAR constructs designed based on variants of CD19-scFv1 showed intermediate binding. Similarly, consistent with the results obtained with scFv-Nluc binding to Raji cells (Fig. 4b), CD19-scFv4-v1-CAR failed to bind CD19-ECD-Nluc, while CD19-scFv4-CAR showed robust binding (Fig. 5b). Collectively, these results demonstrate the potential use of Malibu-Glo assay to select optimal scFvs for the design of CAR constructs.

### Generation and functional evaluation of Malibu-Glo reagents targeting different tumor antigens

**Figure 6:**
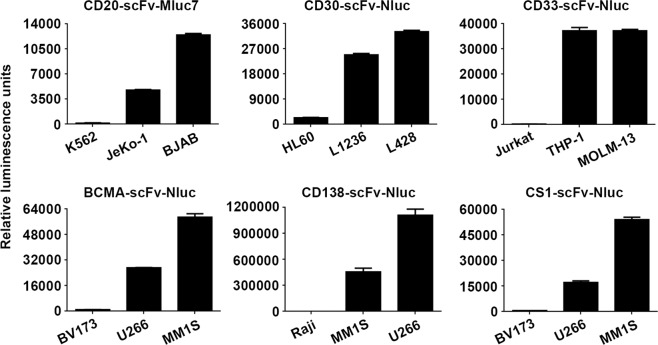
Specific detection of different tumor antigens using Malibu-Glo reagents. The indicated cell lines were incubated with the Malibu-Glo reagents incorporating scFvs targeting CD20, CD30, CD33, BCMA, CD138 and CS1 for 45 minutes on ice. After washing steps, cell bound luminescence was measured.

To extend the Malibu-Glo assay to other antigens for treatment of hematologic malignancies and solid tumors, we generated Malibu-Glo reagents based on scFvs targeting CD20, CD30, CD33, BCMA, CD138 and CS1. The binding ability of the different scFv-Nluc fusion proteins was tested using cell lines that are known to express their respective target antigens (Fig. 6). Thus, CD20-scFv-Nluc Malibu-Glo reagent was tested using JeKo-1 (mantle cell lymphoma) and BJAB (Burkitt lymphoma) cell lines that are known to express CD20, while CD30-scFv-Nluc Malibu-Glo reagent was tested using Hodgkin’s lymphoma derived cell lines L428 and L1236 that express CD30. Similarly, THP-1 (human acute monocytic leukemia) and MOLM-13 (human acute myeloid leukemia) cell line were used to evaluate the binding of CD33-scFv-Nluc, while MM1S (multiple myeloma) and U266 (multiple myeloma) cell lines were used to assess the binding of BCMA-scFv-Nluc, CD138-scFv-NLuc and CS1-Nluc Malibu-Glo reagents. In each case, we used a cell line lacking the desired antigen to determine nonspecific binding. Thus, we used K562, HL60, Jurkat and BV173 as negative control cell lines for checking the binding of Malibu-Glo reagents targeting CD20, CD30, CD33 and BCMA, respectively. As shown in Fig. 6a-f, we observed specific binding of the Malibu-Glo reagents to the expected target antigen-expressing cells, while observing negligible binding to the negative control cell lines. These results demonstrate the feasibility of rapidly generating Malibu-Glo reagent for the detection of any desired cell surface antigen.

### Malibu-Glo assay is a fast and highly sensitive assay for detection of cell surface targets

**Figure 7:**
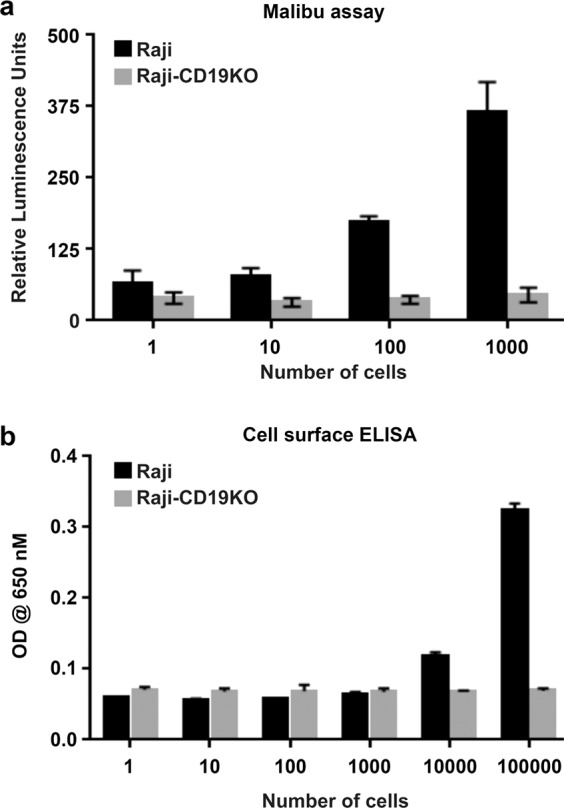
Malibu-Glo assay is a highly sensitive assay. **(a)** Indicated number of Raji cells were mixed with 1× 10^5^ Raji-CD19KO cells following which 100 µl of supernatant containing CD19-scFv fusion protein was added in a final volume of 200 µl. After 45 minutes incubation on ice, cells were washed 5 times prior to the measurement of cell bound luminescence. **(b)** Indicated number of Raji or Raji-CD19KO cells were plated on a poly lysine coated 96-well plate followed by fixation and staining the cells with traditional sandwich ELISA using FMC63 antibody (1 µg/mL).

We next evaluated the sensitivity of the Malibu-Glo assay by incubating supernatants containing CD19-scFv1-Nluc with different number of Raji cells ranging from 1 to 1 × 10^3^ cells. We used 1 × 10^5^ cells of Raji-CD19KO clone A7 to assess the nonspecific binding. Cells were incubated on ice for 45 min, spun, washed five times and luminescence in the cell pellets was measured. As expected, a linear increase in luminescence was observed with increasing number of Raji cells indicating specific binding of CD19-scFv1. Statistically significant differences were observed in wells containing 100 cells and above (Fig. 7a). Further, Malibu-Glo assay showed a linear relation between luminescence values and cell numbers (Supplementary Fig. 4), thereby resulting in a perfect correlation coefficient value (R^2^ = 1). We also compared the sensitivity of Malibu-Glo assay to cell surface ELISA. For this purpose, increasing number of Raji cells were immuno-stained with FMC63, a mouse monoclonal antibody directed against CD19, followed by secondary labeling with a FITC-conjugated anti-mouse antibody. As shown in Fig. 7b, the minimum number of Raji cells that could be detected by cell surface ELISA was 10,000. Therefore, Malibu-Glo assay is at least 100-fold more sensitive than traditional cell-surface ELISA. More studies are needed to compare Malibu-Glo assay with other antigen detection assays.

The standard protocol for the Malibu-Glo assay involves 4 to 5 washing steps to completely eliminate nonspecific binding. However, as specific binding also decreases with every washing step, scFvs with high off rates might dissociate leading to low specific binding^24^. To expand the utility of our assay and to decrease time and labor, we optimized washing steps using CD19-scFv1-Nluc supernatants and cell lines that are positive and negative for the expression of CD19 antigen. As shown in Supplementary Fig. 5, a single wash is sufficient to detect significant differences in the specific binding of CD19-scFv1 to the CD19-expressing Raji and Nalm6 cells over the nonspecific binding to CD19-negative HL60 and Raji-CD19KO cells.

### Malibu-Glo assay using single domain antibodies

**Figure 8:**
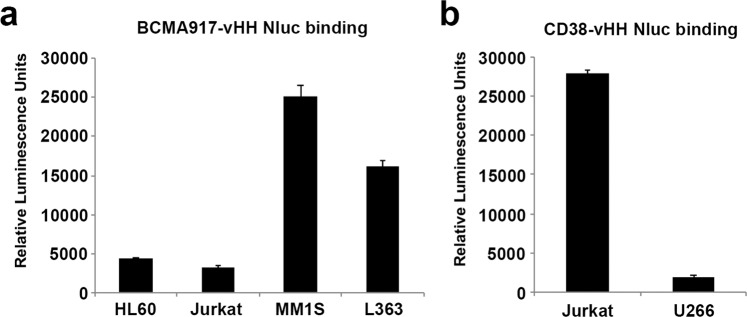
Malibu-Glo assay can be used to evaluate binding of single domain antibodies (VHH). Indicated cells were incubated with BCMA-vHH-Nluc or CD38-scFv-Nluc fusion proteins. After 45 minutes incubation on ice, cells were washed 5 times prior to measurement of cell bound luminescence.

Historically, chimeric antigen receptors have been engineered using scFv as the antigen binding domain. Alternatively, single domain antibodies can be used to generate CAR. To test whether the Malibu-Glo Assay can be extended to heavy chain only single domain antibodies (vHH), we generated Nluc fusions of vHH domains targeting BCMA and CD38 and expressed them in 293FT cells as described earlier. Malibu-Glo assay was performed to evaluate the binding ability of vHH-Nluc fusion proteins. BCMA-vHH-Nluc fusion protein showed significant binding to BCMA-expressing Myeloma cell lines (MM1S and L363), when compared with BCMA-negative HL60 and Jurkat cell lines (Fig. 8). Similarly, Jurkat cells, which express CD38, bound CD38-vHH-Nluc fusion protein, but failed to bind U266 cells, which lack CD38 expression. Thus, Malibu-Glo assay can also be used to screen single domain antibodies to identify potential binders.

### Malibu-Glo assay using non-scFv scaffold proteins

**Figure 9:**
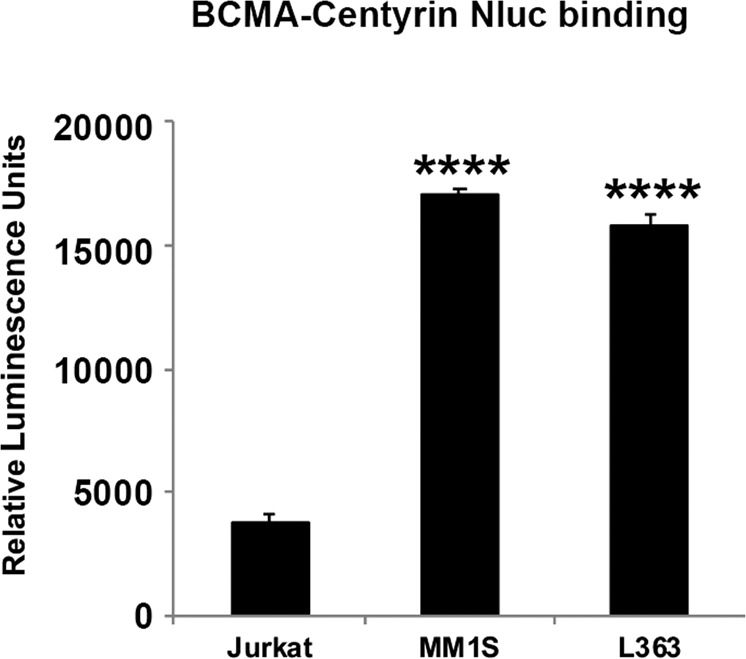
Malibu-Glo assay can be used to evaluate binding of non-scFv antigen binding proteins. Indicated cell lines were incubated with 100 µl of supernatants containing BCMA-Centryin fusion protein for 45 minutes on ice. Cell bound luminescence was measured after washing steps. Statistically significant differences are shown by asterisks (****) at a level of *P* < 0.0001.

In an effort to decrease immunogenicity of non-human scFv derived CAR, which leads to rejection and limited persistence, non-scFv based CARs are being developed using scaffold proteins derived from fibronectin domains, such as centyrins and adnectins^25–27^. To test whether reliable detection of these antigen binding scaffolds can be obtained using the Malibu-Glo Assay, we generated Nluc fusion construct of a centyrin targeting BCMA. We transiently transfected 293FT cells and evaluated the binding of the supernatants to cells expressing BCMA. As expected, BCMA-centyrin selectively bound BCMA-expressing multiple myeloma cells MM1S and L363, but not BCMA-negative Jurkat cells (Fig. 9). Collectively, these results demonstrate that any antigen binding moiety can be fused to a marine luciferase to develop a Malibu-Glo reagent for reliable detection of its target antigen.

## Discussion

Antibody based immunotherapies and chimeric antigen receptor engineered T cell therapies are emerging as promising approaches for treating cancer. The success of these approaches depends on the expression level of the target antigen on tumor cell surface^11,12^ and the relative binding ability of the antigen binding domain of the immunotherapeutic agent^10^. A fast, economical and convenient method is needed for the selection of an immunotherapeutic agent with maximal on-target effect and minimal off-target toxicity.

Currently available methods for antigen-detection, such as western blotting and flow cytometry, are time consuming, labor intensive, require purified reagents and/or expensive equipment. Some biophysical methods (*e.g*., surface plasma resonance) and immunological assays to detect cell surface antigen require purification of either antigen or antibody. Purification procedures, however, can potentially lead to loss of conformational epitopes that are of functional importance. Because of these limitations, it is ideal to detect cell surface antigens in their native context for which flow cytometry still remains the method of choice. However, flow cytometry is time- and labor-intensive, requires expensive equipment and purchase of fluorochrome-conjugated primary or secondary antibodies.

To develop an alternative approach to detect antigens on tumor cell surface, we genetically tagged scFv of antibodies to different marine luciferases. Most previous attempts to generate antibody-luciferase fusion proteins employed large molecular weight luciferases. We used marine luciferases as the fusion partner due to their small size and favorable luminescence characteristics^20^. Other groups have fused marine luciferases to antibody fragments using *E. coli* or phage expression system, where optimal folding and glycosylation of antibody fragments is unpredictable^28,29^. Further, antibody-luciferase fusion proteins need to be purified from interfering bacterial contaminants prior to assessing expression levels or binding to mammalian cells. Additionally, expression levels of antibodies in bacteria may not correlate with their expression levels in mammalian cells. Our assay circumvents these limitations as both expression and binding studies are carried out in mammalian cells, thus saving time and labor to purify antibodies.

Conventional antibody based methods for cell surface antigen detection usually require a secondary detection agent or chemical conjugation of the purified antibody with a fluorophore or a marker enzyme. However, chemical conjugation of antibodies results in their partial inactivation and conjugate heterogeneity, which affects the specificity and sensitivity. This is obviated in the Malibu-Glo reagent as the antigen binding module (e.g., scFv) is coupled directly to the detection module (e.g., Nluc). Thus, the Malibu-Glo reagent has several advantages over antibody-enzyme conjugates obtained using chemical synthesis, including homogenous composition, 1:1 stoichiometry and high sensitivity.

Flow cytometry based detection of surface antigens can be time consuming when cell numbers are limiting, as the rate of sample acquisition is inversely proportional to sample concentrations. For an optimal flow experiment, a minimum of 50–100 K cells are needed. In instances where cell surface antigen detection is required using limited number of patient-derived cells, the Malibu-Glo assay would be advantageous as antigen can be detected even with 100 cells. Furthermore, flow cytometry often demands blocking prior to staining to reduce high nonspecific fluorescence caused by the binding to Fc receptors on cells. Such nonspecific binding is eliminated in our assay as the Malibu-Glo reagent lacks any Fc region.

AlphaLISA is another method to detect a cell-surface antigen in its native conformation. However, the use of AlphaLISA to detect cell membrane bound antigens requires optimization of cell lysis buffer for every antigen^30^. Further, proprietary donor and acceptor beads have to be purchased for conjugating the capture and detection antibody, respectively. Premade kits are available only for few antigens and are expensive. For sandwich AlphaLISA, two antibodies binding to spatially distinct epitopes are required. In comparison, Malibu assay provides a simple, sensitive and cost-effective approach to detect cell surface antigen that is easily adaptable to any lab.

One potential application of the Malibu-Glo assay is in the selection and optimization of scFvs. We demonstrate that the Malibu-Glo assay offers a simple, sensitive, rapid, cost-effective method to simultaneously evaluate the relative expression and binding affinity of scFvs to cell surface targets in a single-step assay format, thereby allowing elimination of weakly expressed scFvs at early stages of antibody optimization.

Successful design of CAR from rapidly growing list of antibodies relies on selection of scFvs with optimal expression and binding characteristics. While it is obvious that the target specificity of a CAR is derived from its antigen binding domain, the rules to select a specific epitope or an ideal antigen binding domain are not obvious. Antibodies may not be available for all antigens and available antibodies may not have undergone prior preclinical or clinical evaluation. On the contrary, for established targets, several antibodies might be available and there is currently no simple, sensitive and rapid method to screen for an optimal scFv fragment for incorporation into CAR construct for further development.

We tested whether expression and target binding of scFvs evaluated using the Malibu-Glo assay would be predictive of the expression level of the corresponding CAR and its binding to the cognate antigen. We demonstrate that the expression level (assessed by MYC staining, Fig. 5a) and the antigen binding ability (assessed by Topanga Assay, Fig. 5b) of a CAR corresponds closely to the expression level and the antigen-binding-ability of the scFv comprising its antigen-binding domain. Indeed, all tested humanized scFvs variants, when incorporated in a CAR, showed expression and target binding strikingly similar to that of the parental scFv. Thus¸ the Malibu-Glo assay is a powerful approach to quickly identify an antigen binding module (e.g., an scFv) with desirable expression and antigen-binding characteristics for incorporation into a CAR. By eliminating scFv with poor expression and binding characteristics at an early stage, the Malibu-Glo assay can streamline the process of CAR construction and potentially eliminate the need for constructing and testing of a large number of CAR constructs against a specific antigen. The assay will also allow construction and testing of CARs against multiple targets in relatively short time to accelerate the pace of preclinical evaluation.

The utility of the Malibu-Glo assay, however, is not limited to selection and optimization of scFvs. Besides scFvs, we have generated Malibu-Glo reagents based on single domain antibodies (vHH) and centyrins. In all cases, tagging with the marine luciferases neither affected the binding of the fusion proteins to their target antigens nor was the luciferase activity compromised, thus demonstrating the versatility of the approach.

Finally, another application of the Malibu-Glo assay is in the identification of cell surface proteins. Although a number of genomic and proteomic approaches are available for the identification of cancer cell specific proteins, they cannot distinguish between the intracellular versus cell surface targets. We believe that the Malibu-Glo assay offers a simple, rapid and powerful tool to identify novel cell surface proteins for biomarker analysis and development of targeted therapies.

In summary, we have developed a fast, simple, economical and efficient assay for detection of cell surface antigens with potential applications in development of antibody and cell therapy products. The Malibu-Glo assay can be a versatile tool to screen for therapeutic antibodies with optimal expression and binding characteristics in the early stages of antibody development projects and to guide the choice of scFvs for incorporation into CAR constructs. The Malibu-Glo assay, however, is not limited to scFv alone and can be extended to bi-specific antibodies, single domain antibodies and other scaffold proteins. Fusion of marine luciferases to other clinically important binding domains and therapeutic proteins can have broad applicability in various areas of biomedicine.

## Materials and Methods

### Cell lines and reagents

Raji, Jurkat, HL-60, JeKo-1, BJAB, RS411 and K562 cell lines were obtained from ATCC and cultured as per the instructions provided. JNG cell line (**J**urkat cells engineered with a **N**FAT-dependent E**G**FP reporter gene) was kindly provided by Dr. Arthur Weiss (University of California, San Francisco, CA, USA). Nalm6 and BV173 cell lines were generous gifts from Dr. Markus Muschen (Children Hospital, Los Angeles, CA, USA). MM1S, U266, THP-1 and MOLM-13 cell lines were kindly provided by Drs. Alan Lichtenstein (Veterans affairs hospital, Los Angeles, CA, USA), Gregor Adams (University of Southern California, Los Angeles), Jae Jung (University of Southern California, Los Angeles) and Li Ling (City of hope, Los Angeles, CA, USA) respectively. L428 and L1236 cell lines were kind gifts from Dr. Markus Mapara (Columbia University Medical Center, NY, USA). For lentiviral production, 293FT cells were purchased from ThermoFisher (Cat #R70007) and maintained as recommended. Polyethylene amine (PEI) was purchased from Polysciences Inc (Cat #24765-1). Polybrene (Cat #107689) and coelenterazine (Cat #303) were obtained from Sigma and Nano light technology, respectively. Cell culture lysis 5X reagent was purchased from Promega (Cat #E153A).

### Generation of Malibu-Glo reagent (scFv-luciferase fusion) expression constructs

Codon optimized cDNA sequences encoding signal peptide deleted variants of different marine luciferases (Nluc, Gluc, Mluc7 and Tluc16) were fused in frame with scFvs and other antigen binding domains^31^ targeting different target antigens and cloned into pLenti-EF1α expression vector using standard molecular biology techniques. An intervening short Gly-Gly-Ser-Gly (GGSG) flexible linker was included between scFv and luciferase sequences. The variable light (vL) and variable heavy (vH) chains of scFvs were also separated by a GGSG linker. These constructs also consisted of a T2A ribosomal skip sequence followed by a puromycin resistance gene (PAC, Puromycin Acetyltransferase). A synthetic cassette 4xFLAG-2xStreptagII-8xHis carrying 4 copies of a FLAG epitope tag, 2 copies of a Strep-tagII and 8 copies of histidine tag was cloned in frame and downstream of the luciferase genes.

### Production of Malibu-Glo reagents

The scFv-Nluc fusion proteins (Malibu-Glo reagents) were produced in 293FT cells by transient transfection of the desired lentiviral constructs using polyethylene amine (PEI). Briefly, 293FT cells were plated overnight in a 100-mm tissue culture plate. For each construct, 10 µg of lentiviral fusion plasmid and 0.25 µg of EGFP plasmid (to assess the transfection efficiency) were suspended in 960 µl of DMEM medium without FBS or antibiotics. To this solution, 21 µl of PEI solution (1 mg/ml) was added dropwise under vortex. After 15 min incubation, the solution containing DNA/PEI complex was added over the 293FT cells. Approximately, 48 hours after transfection supernatants were collected, filtered through a 0.45 µm filter and stored in −80 °C. To assess relative expression of different scFv-Nluc fusions, transient transfections were performed in 24-well plates in duplicate. For each well, 1 µl of PEI was added to 40 µl of DMEM (without FBS or antibiotics) containing 500 ng of the desired scFv-Nluc fusion plasmid, 30 ng of firefly luc plasmid and 20 ng of EGFP plasmid. After 15 min incubation at room temperature, DNA-PEI complex was added to cells in 0.5 ml of DMEM with 10% FBS. After 48 hours, cells were washed gently once with PBS, lysed with 150 µl of 1X cell culture lysis reagent (Promega). Cell lysates were spun at 10,000 rpm for 5 min at 4 °C prior to measurement of luminescence using D-Luciferin as the substrate.

### Generation of Raji clone lacking CD19 using CRISPR/Cas9 technology

Raji cells lacking CD19 were generated by CRISPR/Cas9-mediated gene editing. We employed an all-in-one lentiviral vector pLenti-U6-SFFV-Cas9-2A-Puro that encodes CD19-sgRNA and Cas9 nuclease (Applied Biological Materials Inc.**)**. Lentiviral supernatant was generated by transient transfection using 10 μg of CD19gRNA/Cas9 vector, 7.5 μg of PSPAX2 encoding gag/pol envelope proteins and 2 μg of PLP/VSVG as previously described^18^. Raji cells were transduced with a CD19 gRNA/Cas9 lentivirus in the presence of polybrene (8 μg/ml) by spin-infection (1800 rpm for 45 min at 37 °C). After overnight incubation, cells were washed and suspended in fresh medium with 0.8 μg/ml puromycin. After two weeks, cells were stained with anti-CD19-PE and FACS sorted for the CD19^−ve^ population followed by single cell cloning by limiting dilution.

### Malibu-Glo assay

To establish a cell based binding assay using supernatants containing Malibu-Glo reagent, cells expressing the desired surface targets were counted, spun down at 1300 rpm for 5 min and re-suspended in PBS with 0.5% FBS (2 × 10^6^ cells per ml). For each reaction, 100 µl of target cells were mixed with fusion protein supernatants (100 µl or volumes corresponding to equivalent luminescence units) in duplicate. After 45 min incubation on ice, cells were washed 5 times with 1 ml of ice cold wash buffer (0.5% FBS in PBS) and the pellet was re-suspended in 100 µl wash buffer. Triplicate wells of a white 384-well lumitrac plate were seeded with 30 µl of cells and luminescence was measured using Coelenterazine (CTZ) as substrate^18^.

### Construction of lentiviral based chimeric antigen receptors (CARs) and generation of JNG-CAR cells

We have previously reported the lentiviral vector pLenti-EF1α-FMC63-MYC-BBz-T2A-Pac^18^. Lentiviral vectors encoding different CARs described in the study were generated by swapping the FMC63-scFv with the desired scFv fragments excised from their corresponding scFv-Nluc vector. JNG cells stably expressing different CARs were generated by lentiviral transduction as described previously^18^.

### Statistical analysis

Two-tailed unpaired Student *t* test was used to test for differences between 2 groups using GraphPad Prism 5 software. Differences with a *P* ≤ 0.05 were considered statistically significant.

## Supplementary information

Supplementary information
