# Supplementary material for: A Fast and Sensitive Luciferase-based Assay for Antibody Engineering and Design of Chimeric Antigen Receptors
Source: Sci Rep. 2020 Feb 11;10:2318. doi: 10.1038/s41598-020-59099-9 (PMC7012821; doi:10.1038/s41598-020-59099-9)
Supplement: Supplementary file 1 — Supplementary information [file 41598_2020_59099_MOESM1_ESM.docx]

**Supplementary Material**

**A Fast and Sensitive Luciferase-based Assay for Antibody Engineering and Design of Chimeric Antigen Receptors**

Venkatesh Natarajan*, Ramakrishnan Gopalakrishnan*, Hittu Matta*, Sunju Choi*, Songjie Gong, Alberto Jeronimo, Pooja Smruthi Keerthipati, Anthony Morales, Harishwar Venkatesh and Preet M Chaudhary^#^

*These authors contributed equally to this work.

Jane Anne Nohl Division of Hematology and Center for the Study of Blood Diseases, University of Southern California, Keck School of Medicine, Los Angeles, California, United States of America.

^#^**Corresponding author:** Preet M. Chaudhary, M.D., Ph.D.

**
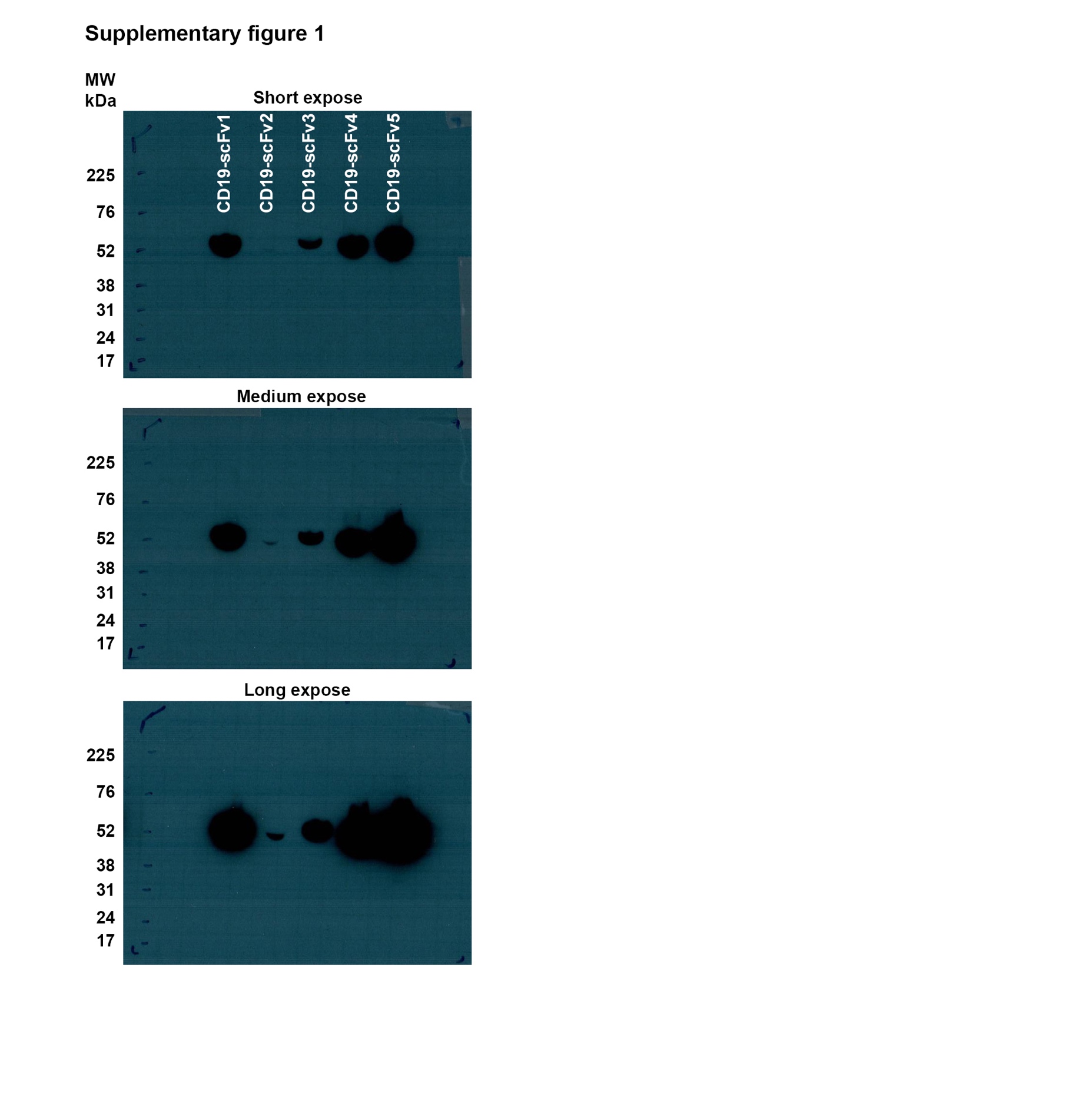
**

**Supplementary Figure 1. Western blot analysis of expression of different CD19-scFv-Nluc fusion proteins.** 10 µl of indicated supernatants were separated by 10% SDS-PAGE, followed by western blotting using a Flag antibody (M2-HRP; Sigma).


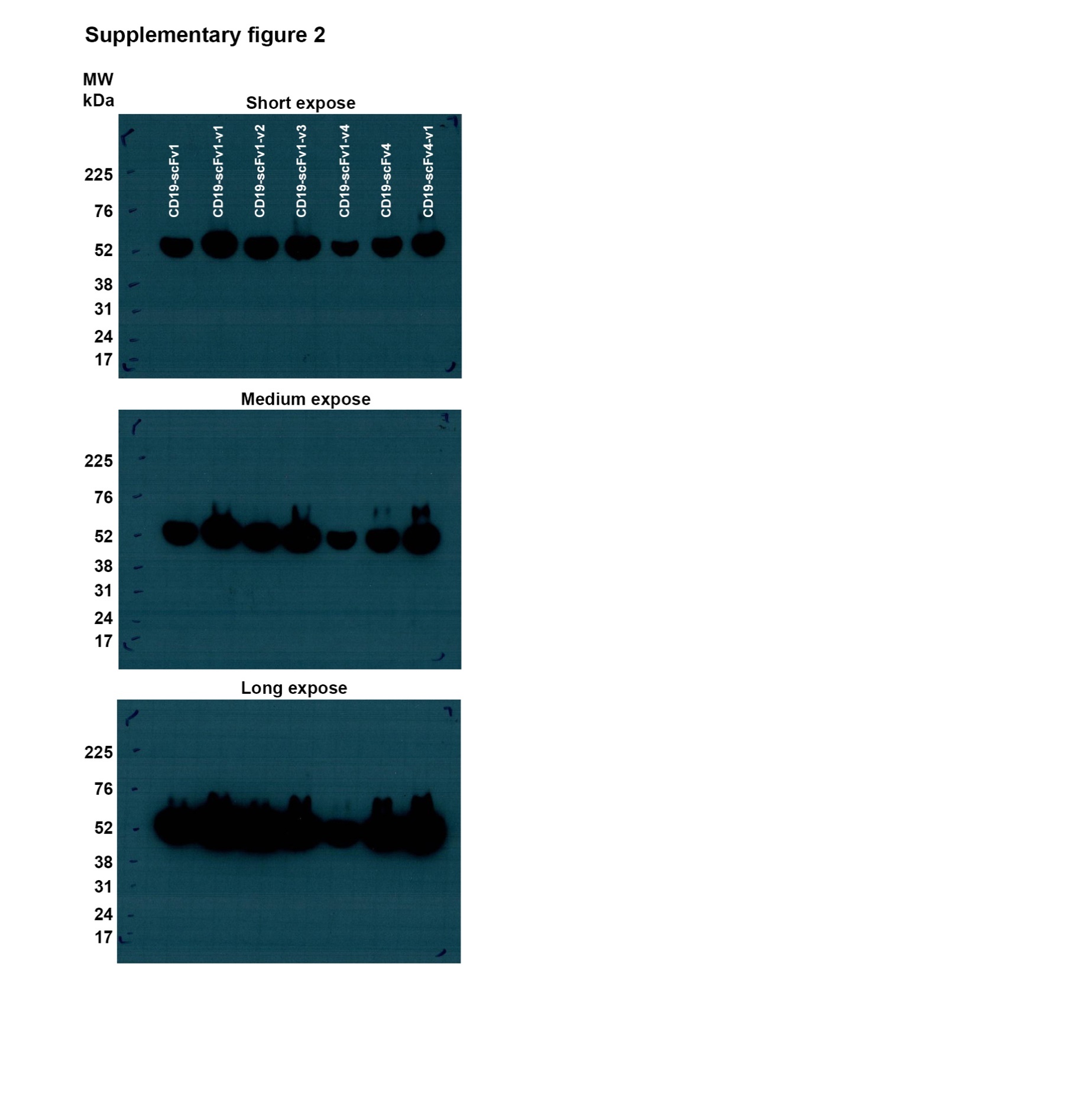


**Supplementary Figure 2. Western blot analysis of expression of different CD9-scFv-NLuc variants.** 10 µl of the indicated supernatants were separated by 10% SDS-PAGE followed by western blotting using a Flag antibody (M2-HRP; Sigma).


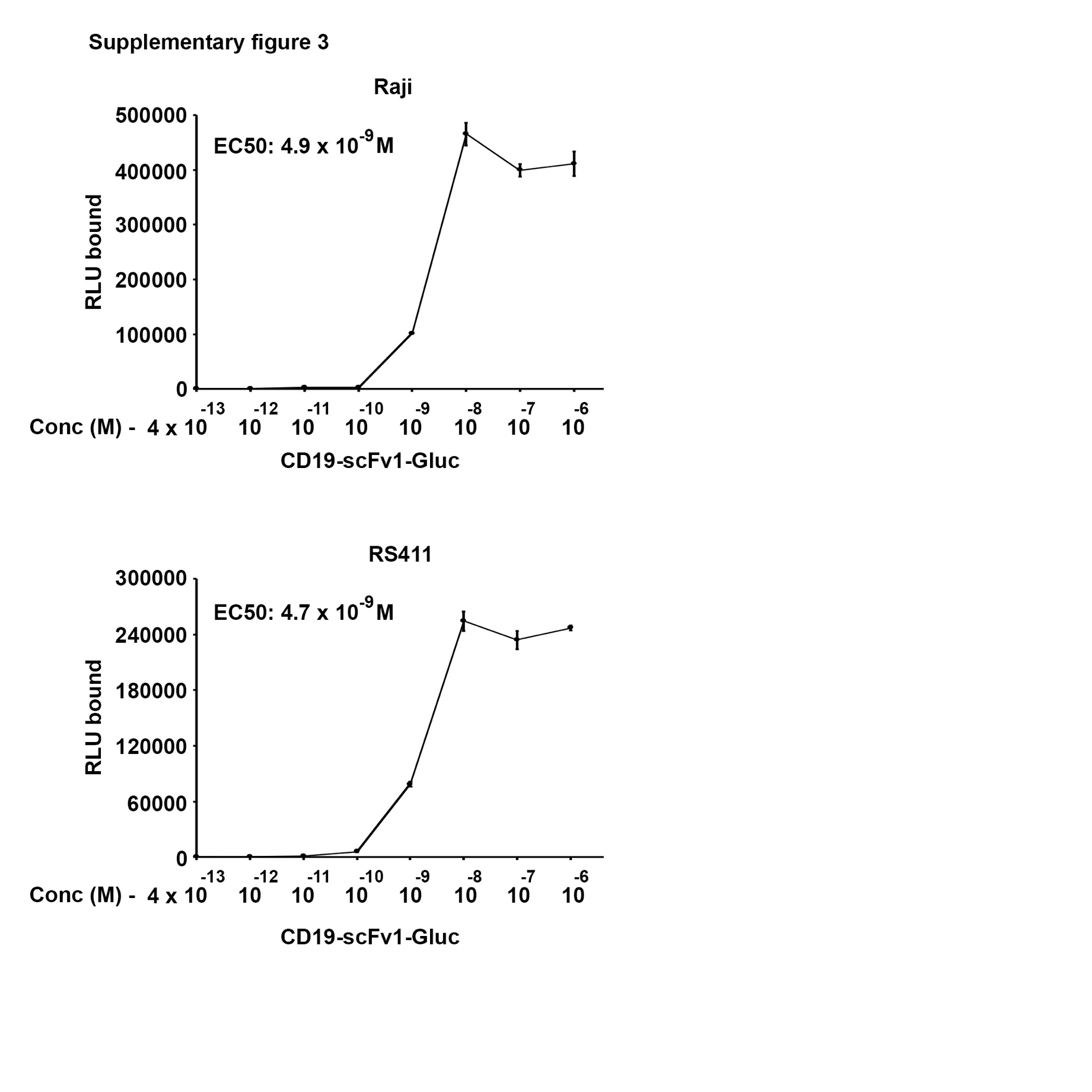


**Supplementary Figure 3. Determination of EC50 of purified CD19-scFv-Gluc using Malibu-Glo Assay.** Raji cells and RS411 cells (1 x 10^4^) were incubated with increasing amount of purified CD19-scFv-Gluc for 45 minutes on ice. Cells were washed 6 times prior to measurement of cell bound luminescence, which was used to measure EC50 using variable slope model. A representative of two independent experiments were shown.


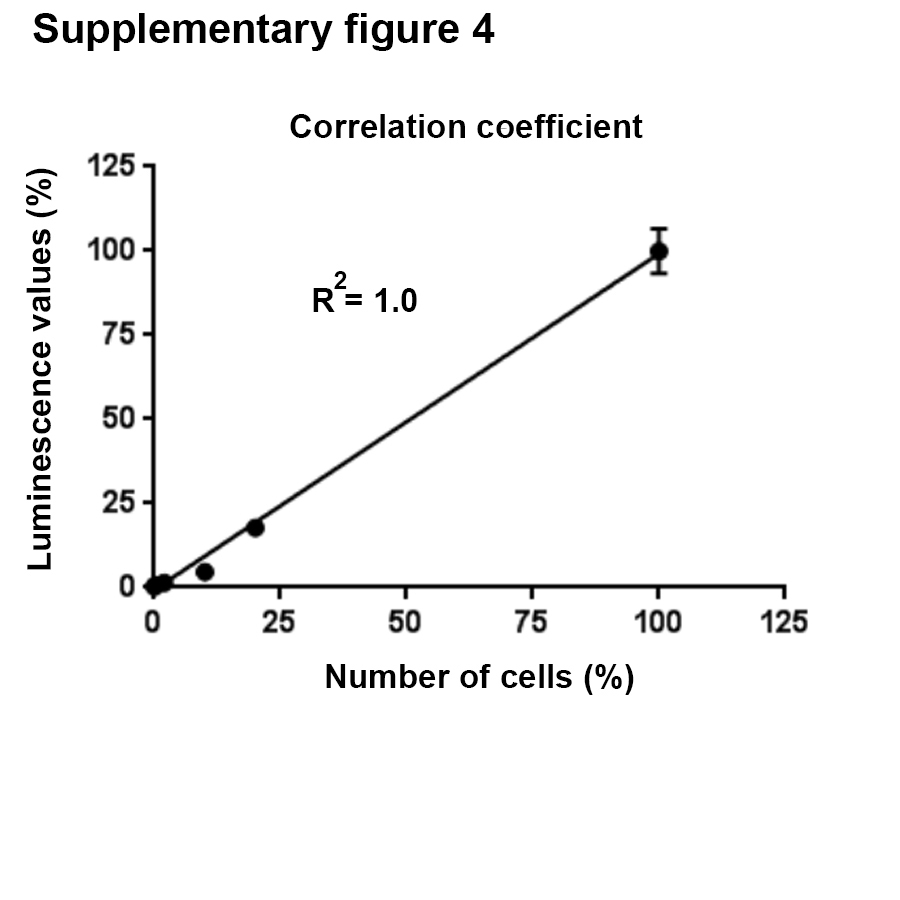


**Supplementary Figure 4. Linear increase in luminescence over a wide range of cell numbers.** Both the number of Raji cells plated and luminescence values detected were converted into percentage by dividing the individual values with the maximum number of cells plated (50000) or the luminescence values from the wells with maximum cell number. R^2^ = Correlation coefficient

**
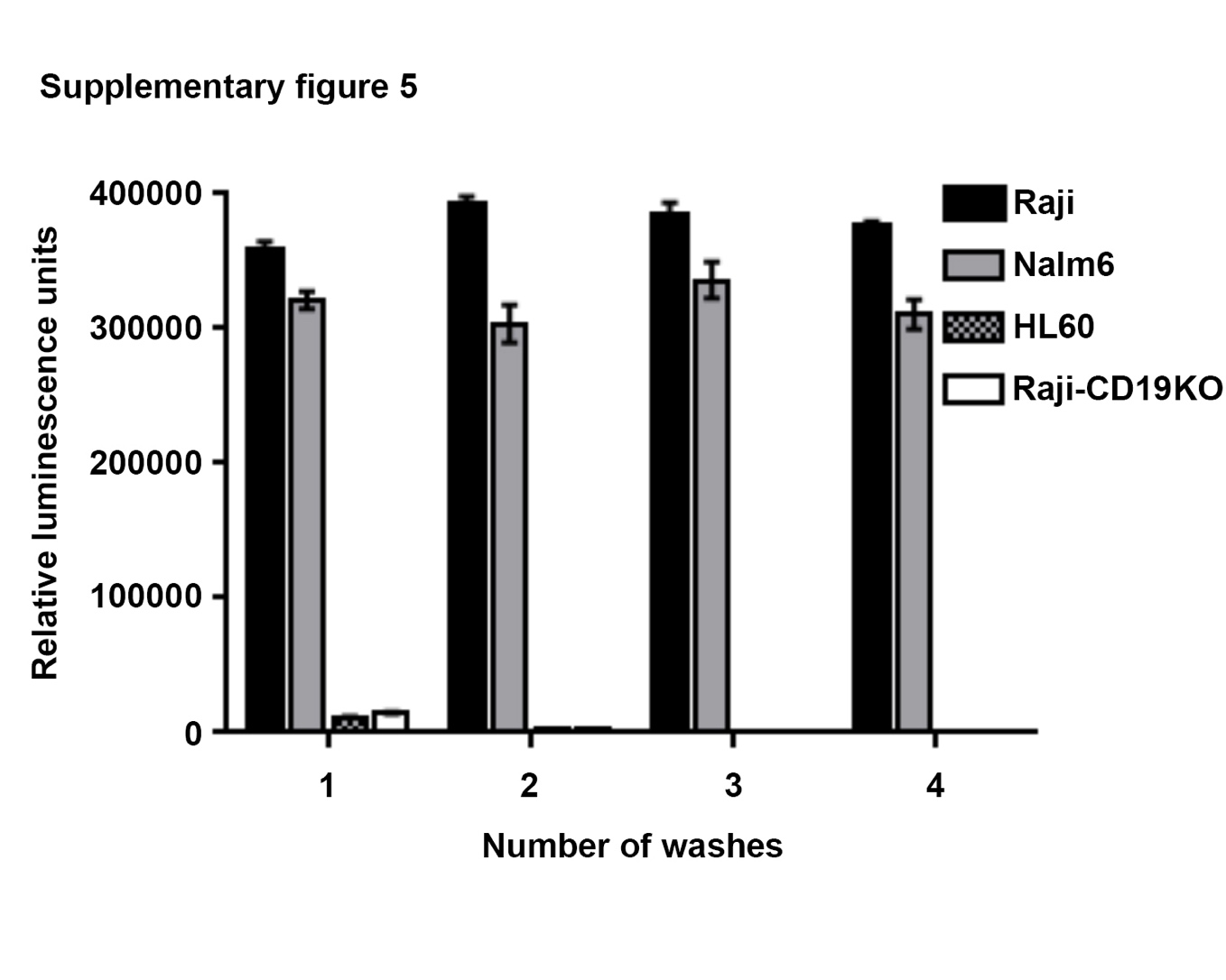
Supplementary Figure 5. Optimization of washing steps**. Indicated cell lines were incubated with 100 µl supernatant containing CD19-scFv-Nluc fusion protein for 45 minutes on ice. After indicated number of washing steps cell bound luminescence was measured. A representative of two independent experiments is shown.
